# Zymosan promotes proliferation, *Candida albicans* adhesion and IL-1β production of oral squamous cell carcinoma in vitro

**DOI:** 10.1186/s13027-020-00315-6

**Published:** 2020-07-31

**Authors:** Xu Chen, Qingqiong Luo, Jieying Ding, Meng Yang, Ruiyang Zhang, Fuxiang Chen

**Affiliations:** 1 Department of Clinical Immunology, Shanghai Ninth People’s Hospital, Shanghai Jiao Tong University School of Medicine (SJTUSM), Shanghai, 200011 China; 2 Faculty of Medical Laboratory Science, Shanghai Jiao Tong University School of Medicine (SJTUSM), Shanghai, 200025 China

**Keywords:** Zymosan, *Candida albicans*, Proliferation, Oral cancer, Oral squamous cell carcinoma

## Abstract

Oral squamous cell carcinoma (OSCC) is the most common type of head and neck squamous cell carcinoma (HNSCC), and the effect of zymosan (ZYM), a component of the yeast cell wall, on oral cancer remains unclear. The CCK-8 proliferation assay was performed to evaluate the effect of ZYM on the proliferation of the OSCC cell lines WSU-HN4, WSU-HN6 and CAL27, and the potential mechanism was explored by quantitative real-time PCR, immunofluorescence assay and western blot. A cell adhesion assay was conducted to determine the adhesion of *Candida albicans* to OSCC cells, and the expression of related genes, including *TLR2*, *MyD88*, *NLRP3*, *ASC*, *Caspase-1* and *IL-1β*, and proteins, including TLR2, MyD88, NF-κB p65, p-NF-κB p65 and E-cadherin was determined. Additionally, the pro-inflammatory cytokines including IL-6, IL-8, TNF-α and IL-1β produced by OSCC cells were detected using a chemiluminescence immunoassay (CLIA). In the current study, the CCK-8 assay showed that ZYM promoted the proliferation of WSU-HN4, WSU-HN6 and CAL27 cells via the TLR2/MyD88 pathway. The cell adhesion assay showed that the number of *C. albicans* cells per field significantly increased in ZYM-treated OSCC cells compared to controls. When treated with ZYM, OSCC cells secreted significantly more pro-inflammatory cytokine IL-1β, which could enhance inflammation in oral cancer microenvironment. In conclusion, ZYM from the fungal cell wall promotes the proliferation, *C. albicans* adhesion and IL-1β production in OSCC, as demonstrated by in vitro experiments.

## Introduction

Oral squamous cell carcinoma (OSCC) is the most common type of head and neck squamous cell carcinoma (HNSCC), which is the sixth leading cancer type worldwide [1]. The most common treatment for oral cancers involves radical surgery and chemotherapy, including 5-fluorouracil and cisplatin. However, the 5-year survival rate among patients with OSCC is low because of the poor prognosis, such as the occurrence of lymph node metastasis and local recurrence [2]. Recently, imbalanced microbiota or specific microbes have been found to play an indispensable role in tumour initiation, progression and even chemoresistance [3–6]. For example, *Helicobacter pylori* plays a role in inducing or promoting gastric cancer [7], and different microorganisms, such as *Fusobacterium* sp., *Porphyromonas gingivalis* and *Candida albicans*, have been reported in oral cancers [8–10].

It has been observed that oral carriage of *C. albicans* is higher in patients with OSCC or leukoplakia than in those without oral pathology [11, 12], but the association between *C. albicans* and oral cancer remains ambiguous. Several reports have proposed different potential pathways by which *C. albicans* initiates or promotes carcinogenesis, such as inducing inflammation [13, 14]. Triggering of inflammation by inducing TNF-α and IL-8 or production of carcinogenic by-products such as nitrosamine and N-nitrosobenzylmethylamine (NBMA) by *C. albicans* could be a risk factor for cancer [15]. Therefore, it is important to investigate the interaction between microorganisms and host cells. The adhesion of microorganisms to host cells has been reported to be the first step for bacteria or fungi to influence host cells [8]. However, the adhesion of *C. albicans* to OSCC cells remains unclear.

Toll-like receptors (TLRs) and other pattern recognition receptors (PRRs) play a significant role in the recognition of pathogens and triggering of innate immune responses, and a group of molecules can act as pathogen-associated molecular patterns (PAMPs), such as glucans, which are recognized by PRRs [16]. Moreover, the OSCC cells have been found to express different TLRs, including TLR3 and TLR4 [17]. Activation of TLRs usually induces recruitment of the adapter molecule myeloid differentiation primary response protein 88 (MyD88), and activation of TLR/MyD88 typically leads to the activation of NF-κB, which has been linked to the progression of tumours [18]. However, the role of TLR expressed on OSCC cells in the interaction with microbial components needs to be investigated. This study aimed to define the effect of zymosan (a component of the fungal cell wall, ZYM), which is a glucan with repeating glucose units connected by β-1,3-glycosidic linkages, on oral cancer by investigating the proliferation and cytokine production of OSCC cells and *C. albicans* adhesion to cell lines.

## Materials and methods

### Cell lines and reagents

In this study, the human OSCC cell lines WSU-HN4, WSU-HN6 and CAL27 were cultured in Dulbecco’s modified Eagle’s medium (DMEM) supplemented with 10% fetal bovine serum (Gibco, New York, NY, USA) and 1% penicillin-streptomycin. All cells were incubated in a humidified atmosphere containing 5% CO_2_ at 37 °C. Zymosan was purchased from Sangon Biotech (Shanghai, China). *C. albicans* ATCC 90028 was cultured on Sabouraud Dextrose Agar (SDA) at 37 °C in an incubator containing 5% CO_2_.

### CCK-8 cell viability assay

The cell viability assay was performed with CCK-8 (Dojindo, Kumamoto, Japan). OSCC cells (5000 cells/well) were seeded into 96-well plates and cultured for 12–24 h before treatment. The OSCC cells were treated with zymosan (10 μg/ml and 100 μg/ml) or with 100 μL PBS as the control. After 12 h, 24 h or 48 h of treatment, 10 μL of CCK-8 reagent was added, and the optical density was read at 450 nm on a microplate reader (Bio-Rad, Hercules, CA, USA) after incubation for 1.5 h.

### Cell adhesion assay

OSCC cell suspension was seeded onto a coverslip placed in a 24-well plate, which was disinfected by UV light, and the cells were incubated for 24 h to attach to coverslip, and then stimulated by zymosan or PBS for an additional 24 h. The cells were washed with PBS before adding *C. albicans* suspension in PBS, and were further incubated at 37 °C with 5% CO_2_ for 1 h. After incubation, the DMEM with unattached yeast was aspirated, and each well was washed with PBS and then fixed with 95% ethanol for 1 h. After that, the coverslip was removed for Gram staining.

### Cytokine detection

OSCC cells were seeded into a 12-well plate before treatment with PBS or zymosan, and then supernatants were collected to measure the concentrations of IL-1β, IL-6, IL-8 and TNF-α. These cytokines were measured by chemiluminescence immunoassay (CLIA) using IMMULITE®1000 (SIEMENS, Germany) with commercial reagents according to the manufacturer’s instructions.

### Quantitative real-time PCR

Total RNA was extracted using TRIzol reagent (Invitrogen, San Diego, CA, USA), and real-time PCR amplification was carried out by a two-step reaction. First, cDNA was synthesized by GeneAmp® PCR system 9700 (Applied Biosystems) with PrimeScript™ RT Reagent kit (TaKaRa, Shiga, Japan), and then real-time PCR was performed in the 7500 system (Applied Biosystems) with SYBR Premix Ex Taq II (TaKaRa). The experiment was repeated in triplicate on independent occasions. The *GAPDH* gene was used for the normalization of gene expression, and the relative expression of *TLR2*, *MyD88*, *NLRP3*, *ASC*, *Caspase-1* and *IL-1β* was determined using the 2^−ΔΔCt^ method.

### Western blot

OSCC cells treated with zymosan (100 μg/mL) were collected and lysed on ice in RIPA buffer with phosphatase inhibitor (Solarbio, Beijing, China) and phenylmethanesulfonylfluoride (PMSF). After centrifugation, the protein concentration in supernatants was determined by an Enhanced BCA Protein Assay Kit (Beyotime, Haimen, China), and the protein samples were then incubated at 100 °C for 10 min. Equal amounts of total protein were subjected to SDS-polyacrylamide gel electrophoresis (SDS-PAGE) and then electrophoretically transferred onto a polyvinylidene difluoride (PVDF) membrane (Bio-Rad, Hercules, CA, USA). The membrane was blocked in 5% nonfat milk powder in Tris-buffered saline/Tween 20 (TBST) for 1 h at room temperature and incubated with primary antibody overnight at 4 °C (TLR2, ab16894, Abcam; MyD88, ab133739, Abcam; E-cadherin, ab40772, Abcam; NF-κB p65, #8242, Cell Signaling Technology; p-NF-κB, #3033, Cell Signaling Technology and β-actin, A1978, SIGMA) and then incubated with HRP-conjugated secondary antibodies (anti-rabbit IgG from Sigma-Aldrich and anti-mouse IgG from Cell Signaling Technology) at a dilution of 1:5000 for 1 h at room temperature. Protein bands were visualized using High-sig ECL substrate and a Tanon 5200 CE machine (Tanon, Shanghai, China).

### Immunofluorescence assay

The OSCC cells treated with PBS or zymosan and placed on glass slides were fixed with 4% paraformaldehyde for 20 min, permeabilized with 0.1% Triton X-100 for 3 min and blocked with 5% bovine serum albumin for 30 min. The slides were incubated with anti-TLR2 antibody (1:10, Abcam, MA, USA) at 4 °C overnight. Anti-mouse IgG antibody with Alexa Fluor 568 served as secondary antibody (Thermo Fisher Scientific, USA). After washing three times with TBST, the cells were stained with DAPI for 3 min to visualize the nuclei. Images were taken by an inverted microscope equipped with fluorescence optics (Olympus, Osaka, Japan).

### Statistical analysis

The data were displayed as the mean ± standard deviation, and processed by GraphPad Prism 5 (version 5.01, GraphPad Software, CA, USA). Statistical analysis was conducted by SAS 8.2 (SAS Institute Inc., Cary, NC, USA). Two-tailed *P*-value less than 0.05 was considered statistically significant and was indicated with * when *P* < 0.05, ** when *P* < 0.01, *** when *P* < 0.001 and **** when *P* < 0.0001.

## Results

### Zymosan from the fungal cell wall promotes the proliferation of OSCC

**Fig. 1:**
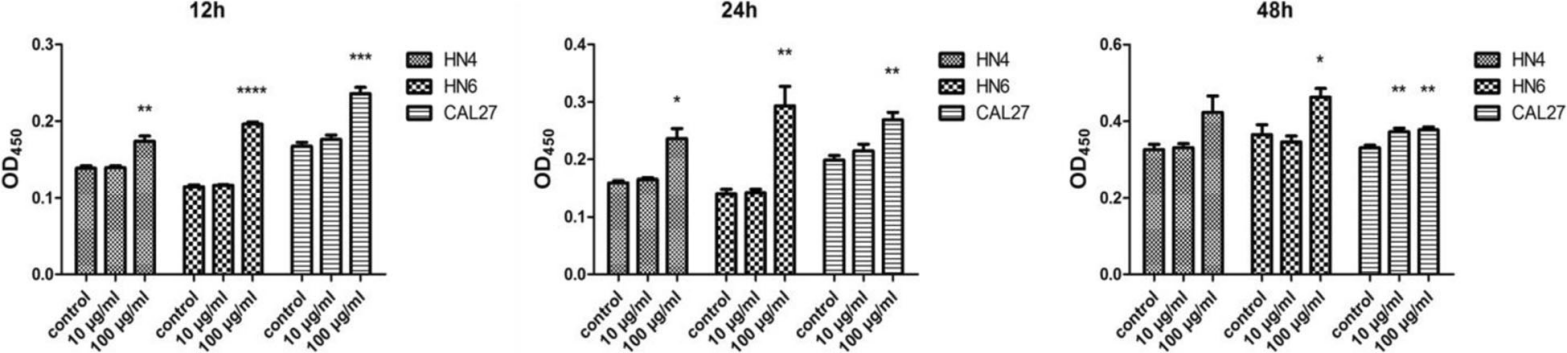
Proliferation of OSCC cells treated with/without zymosan

To investigate the effect of ZYM on oral cancer cells, we cultured OSCC cell lines (WSU-HN4, WSU-HN6 and CAL27) with zymosan at the concentrations of 10 μg/ml and 100 μg/ml for 12 h, 24 h or 48 h. There was no influence on the growth of OSCC cells when the zymosan concentration was 10 μg/ml. However, zymosan promoted the proliferation of OSCC cells in vitro according to the results of the CCK-8 assay when the concentration was 100 μg/ml (Fig. 1). The results indicated that a tumour-promoting effect could be induced by zymosan or other glucan-containing pathogens such as *C. albicans*, at a high concentration.

### The TLR2/MyD88 pathway is involved in the interaction between OSCC and zymosan

**Fig. 2:**
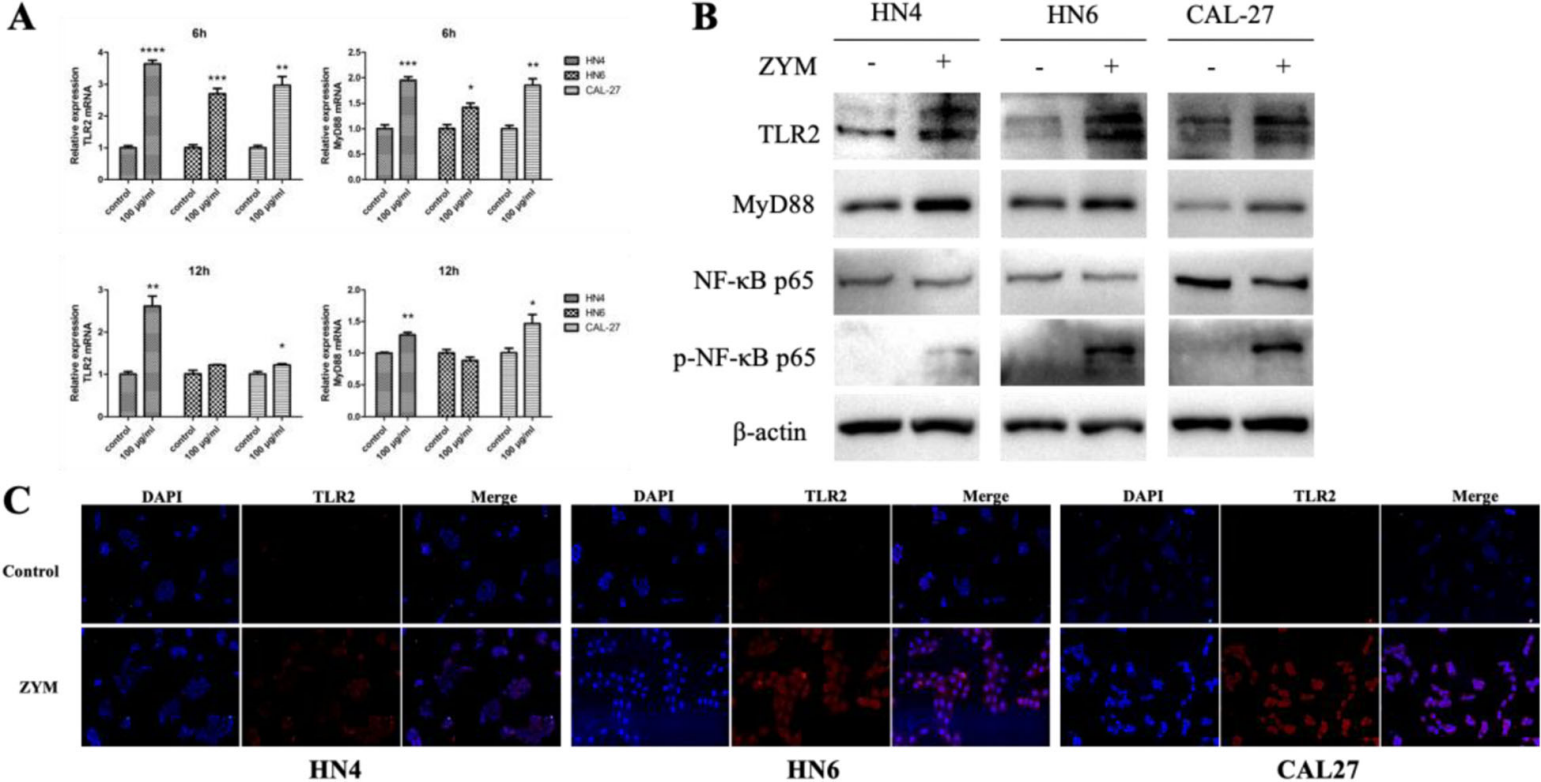
Zymosan activated the TLR2/MyD88/NF-κB pathway in OSCC cells

To define the receptors of zymosan in OSCC cells, the mRNA expression of receptors including TLR2, TLR6, Dectin-1 and CR3 was detected by quantitative real-time PCR (RT-PCR). The results suggested that TLR2 is expressed on OSCC cells and that TLR2 is more highly expressed when the cells were treated with zymosan (Fig. 2a and b). Elevated expression of TLR2 on OSCC cells treated with zymosan was also found by immunofluorescence assay (Fig. 2c). By RT-PCR and western blot, the expression of the downstream adapter molecule MyD88 was also found to be elevated, indicating that the TLR2/MyD88 pathway is involved in the process of stimulation. The activation of NF-κB, which is commonly induced by TLR2/MyD88, has been linked to the progression of tumours. As shown in Fig. 2b, phosphorylated-NF-κB p65 protein levels were significantly increased in OSCC cells treated with zymosan compared with those without zymosan. These results indicated that zymosan could facilitate the proliferation of OSCC in vitro by activating NF-κB via the TLR2/MyD88 pathway.

### Increased adhesion of *C. albicans* onto OSCC treated with zymosan

**Fig. 3:**
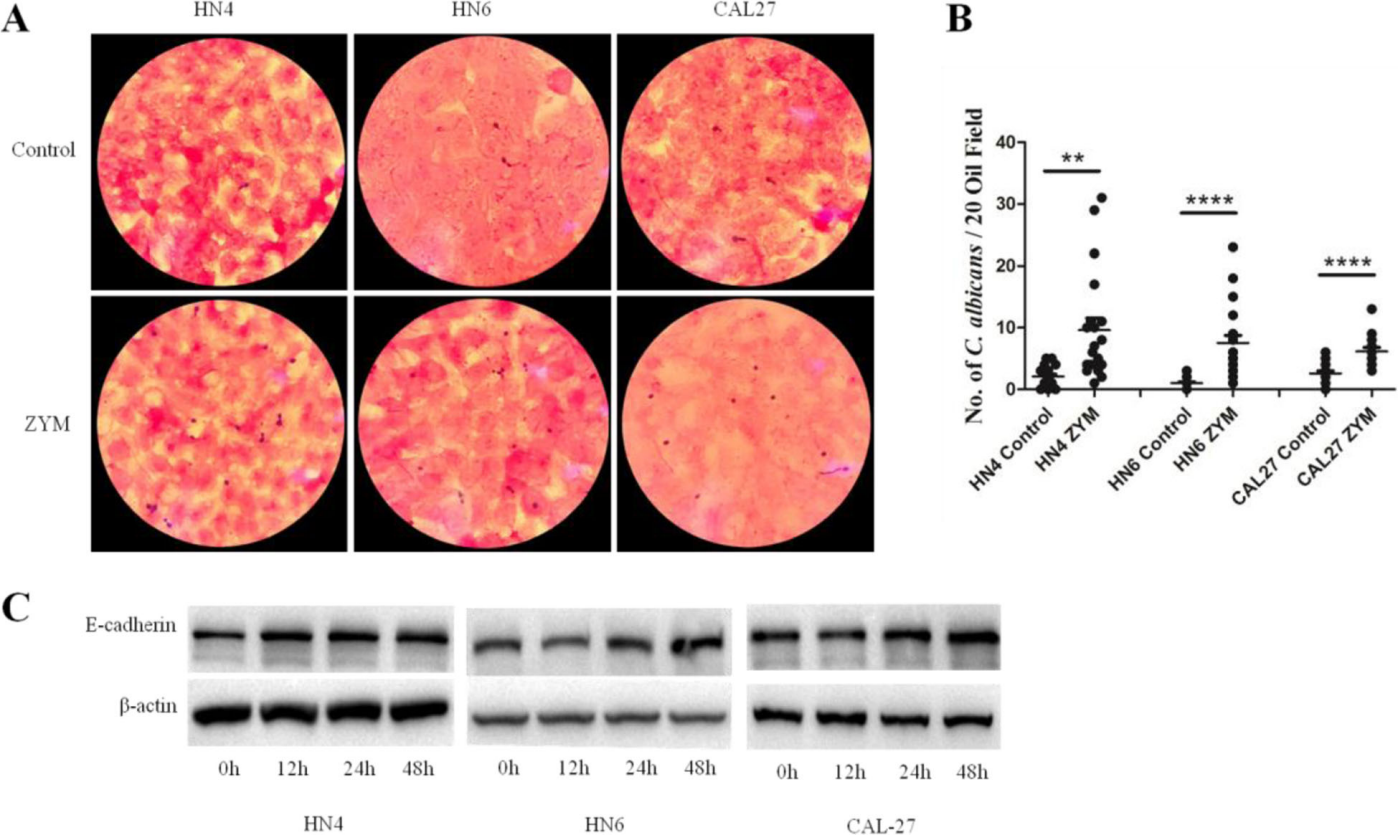
Increased adhesion of *C. albicans* onto zymosan-treated OSCC cells

Clinical observations have indicated an association between *C. albicans* and oral cancers. In the cell adhesion test, the number of *C. albicans* cells per oil field was significantly increased in ZYM-treated OSCC cells compared to controls (Fig. 3a), suggesting increased adhesion of *C. albicans* onto ZYM-treated OSCC cells. After summarizing the total number of *C. albicans* cells from 20 oil fields, a significant increase in *C. albicans* cells was found on the surface of ZYM-treated OSCC cells including WSU-HN4, WSU-HN6 and CAL27 (Fig. 3b). It has been reported that E-cadherin plays an important role in the bacterial adhesion [19]. Thus, we determined the expression of E-cadherin by western blot, and the E-cadherin was more highly expressed in OSCC cells treated with ZYM for 48 h (Fig. 3c).

### Elevated IL-1β production in OSCC cells treated with zymosan is observed

**Fig. 4:**
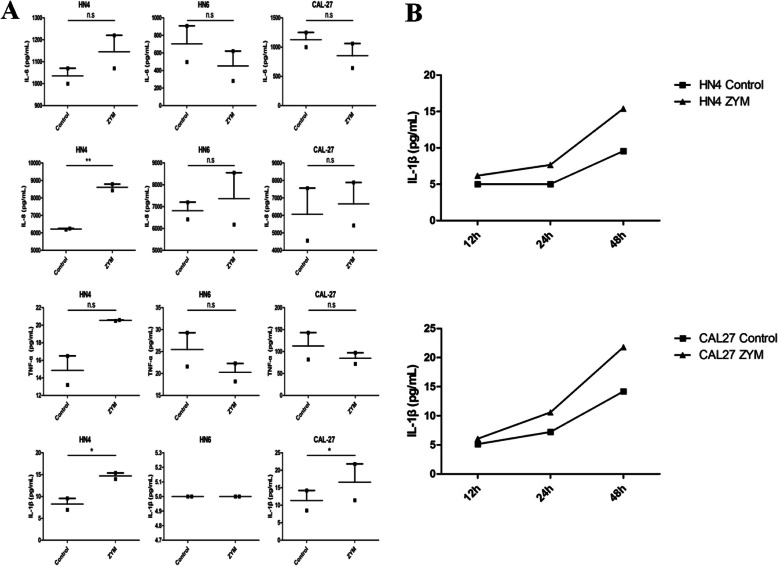
Detection of pro-inflammatory cytokine in the culture medium of OSCC cells

The pro-inflammatory cytokines including IL-6, IL-8, TNF-α and IL-1β in the culture supernatant from WSU-HN4, WSU-HN6 and CAL27 cell lines treated with/without zymosan were detected. By statistical analysis, there was a significant difference in the level of IL-1β produced by ZYM-treated WSU-HN4 (*p* = 0.0482) and ZYM-treated CAL27 cells (*p* = 0.0451) compared with controls (Fig. 4a). However, the concentration of IL-1β from WSU-HN6 cells treated with/without zymosan was below 5 pg/ml. Then, we determined the dynamic level of IL-1β in WSU-HN4 and CAL27 cell lines, and the results showed an increased time-dependent tendency (Fig. 4b). Moreover, when treated with ZYM, OSCC cells secreted significantly more IL-1β, which was indicative of an inflammatory response in the OSCC (Fig. 4b).

### NLRP3 inflammasome is activated in zymosan-treated OSCC cells

**Fig. 5:**
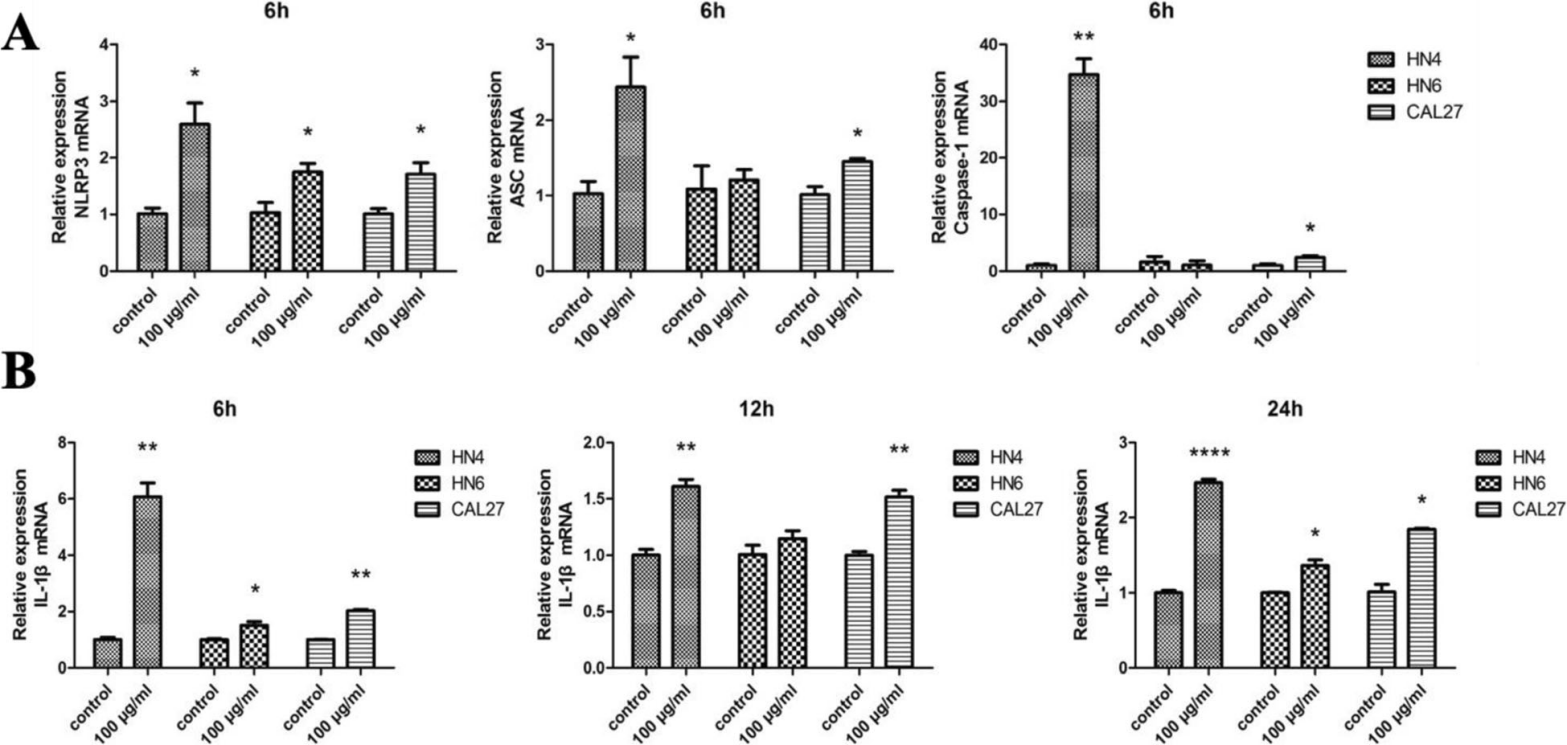
Expression of NLRP3/ASC/Caspase-1/IL-1β in OSCC cells

Activation of the NLRP3 inflammasome is the main path to produce IL-1β, and then we determined the expression of NLRP3, ASC and caspase-1 by RT-PCR. The expression of NLRP3, ASC and caspase-1 was enhanced in WSU-HN4 and CAL27 cells (Fig. 5a). The expression of IL-1β was highly enhanced in OSCC cells at both the mRNA and protein levels (Figs. 4b, 5b). These data suggested that zymosan could trigger the NLRP3 inflammasome and that NLRP3/IL-1β was also activated in OSCC cells when treated with the fungal cell wall component zymosan.

## Discussion

**Fig. 6:**
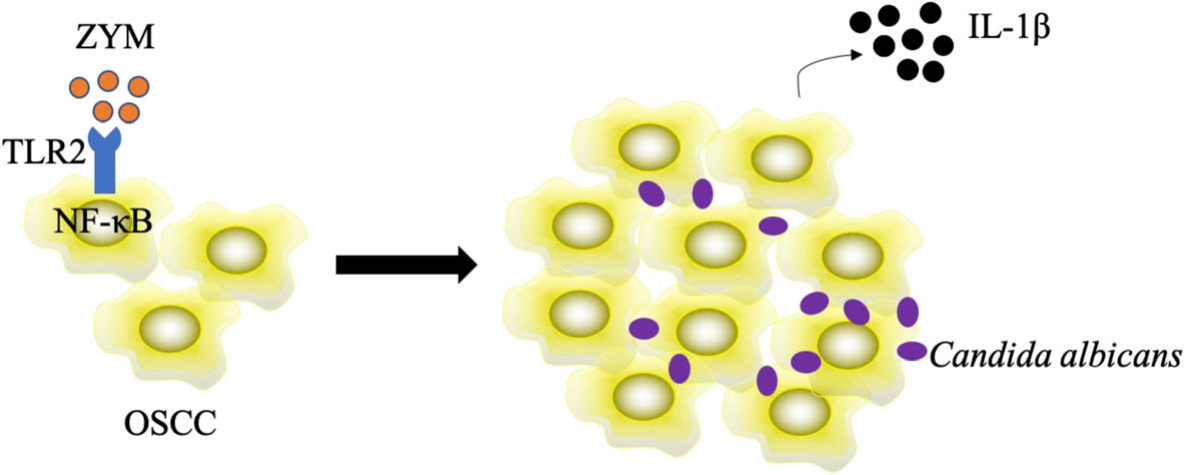
Schematic diagram of this study

In the present study, we found that zymosan from the fungal cell wall promotes the proliferation of OSCC cells via the TLR2/MyD88/NF-κB signaling pathway. In addition, zymosan could promote the expression of E-cadherin to enhance the adhesion of *C. albicans* onto OSCC cells and could further increase IL-1β production by OSCC cells. These results can provide information to better understand the interaction between *C. albicans* and oral cancer (Fig. 6).

As the main type of head and neck squamous cell carcinoma (HNSCC), oral cancer has been a significant clinical problem worldwide. There is a geographic variation in the incidence of oral cancer, and the countries in South Asia are traditionally considered high-occurrence places [20]. High risk factors for oral cancer include heavy smoking, excessive alcohol usage and betel chewing. Furthermore, microbiological infections, such as HPV infection and subsequent inflammation, may also increase the risk of oral cancer, while other microorganisms such as *P*. *gingivalis*, *F*. *nucleatum* and *C. albicans*, play a significant role in the process of oral cancer [21–24]. However, the detailed mechanism linking these microbes and oral cancer remains unknown. Here, we found that zymosan could promote the proliferation of OSCC in vitro via the traditional receptor TLR2. It has been reported that the TLR2-regulated gene signature is associated with tumour growth and that TLR2-dependent inflammation mediates tumour metastasis [25, 26]. Moreover, other TLRs, such as TLR3 and TLR4, are also expressed on OSCC [17, 27]. These results suggested that PMAPs play a pivotal role in the interaction of oral cancer and microbiota and that targeting TLRs in OSCC is a promising therapeutic method. Moreover, an elevated number of *C. albicans* was observed in OSCC cells when treated with zymosan, which implied increased adhesion/interaction between *C. albicans* and OSCC. This experiment could explain the phenomenon that *C. albicans* is higher in patients with OSCC or leukoplakia than in those without oral pathology. E-cadherin was reported to play a significant role in the adhesion of bacteria onto host cells [19], and here, we also detected higher levels of E-cadherin in OSCC cells after ZYM treatment for 48 h, indicative of E-cadherin in the adhesion of *C. albicans* onto OSCC. However, the detailed role of E-cadherin needs to be confirmed in animal models or clinical samples.

Recently, it was reported that *P*. *gingivalis* promoted oral carcinogenesis and aggravated the disturption of fatty acid metabolism, indicating a close association of *P*. *gingivalis*, lipid metabolism and oral carcinogenesis [21]. With the increased attention on microbiology, increasing research has shed light on the interaction between microbiota and cancers. Dejea et al demonstrated a synergistic interaction between two carcinogenic bacteria with different toxins and established the concept of microbial networks in carcinogenesis [28, 29]. As a key element in oral cancers, inflammation plays a significant role in the crosstalk between tumours, immune cells and microorganisms [30], and infections usually triggers inflammatory processes. Here, we found that the fungal cell wall component zymosan was involved in OSCC cells secreting significantly more pro-inflammatory cytokine IL-1β through the NLRP3/IL-1β pathway, indicating an inflammatory response in the OSCC. It is interesting to find that OSCC cells could secrete pro-inflammatory cytokines (such as IL-1β) and be influenced by microbiota or their cell components, which indicates a more complex interaction between cancer cells, immune cells and microbiota in tumour microenvironment. Another report from our lab revealed that NLRP3 inflammasome-activated IL-1β promoted 5-FU resistance in OSCC both in vitro and in vivo [31], and combining these data, it can be inferred that the presence of *C. albicans* in oral cancer may influence the effect of chemotherapy by inducing IL-1β production, which was also a potential target for treating oral cancer.

Despite some interesting findings in the current study, there are still some limitations: 1) all the results were based on in-vitro experiments; 2) the mechanism explored in the current study needs further confirmation by more experiments, such as siRNA transfection; and 3) the mechanism should be verified by animal experiments and clinical human samples.

## Conclusion

In summary, the fungal cell wall component zymosan promoted OSCC proliferation, IL-1β production and *C. albicans* adhesion to OSCC in this study, providing information to better understand the interaction between *C. albicans* and oral cancer.

## Data Availability

All data generated or analyzed during this study are included in this article, and the data is also available from the corresponding author on reasonable request.

## Abbreviations

OSCC: Oral squamous cell carcinoma
HNSCC: Head and neck squamous cell carcinoma
CCK-8: Cell counting kit-8
ZYM: Zymosan
NBMA: N-nitrosobenzylmethylamine
TLR: Toll-like receptor
PRR: Pattern recognition receptor
DMEM: Dulbecco’s modified Eagle’s medium
SDA: Sabouraud dextrose agar
PMSF: Phenylmethanesulfonylfluoride
SDS-PAGE: Sodium dodecyl sulfate-polyacrylamide gel electrophoresis
PVDF: Polyvinylidene difluoride
TBST: Tris-buffered saline/Tween 20
MyD88: Myeloid differentiation primary response protein 88
NLRP3: NACHT, LRR and PYD domains-containing protein 3
IL: Interleukin
5-FU: 5-Fluorouracil
PCR: Polymerase chain reaction
